# Research on Visualization and Error Compensation of Demolition Robot Attachment Changing

**DOI:** 10.3390/s20082428

**Published:** 2020-04-24

**Authors:** Qian Deng, Shuliang Zou, Hongbin Chen, Weixiong Duan

**Affiliations:** 1 College of Mechanical Engineering, University of South China, Hengyang 421001, China; dengqian@usc.edu.cn (Q.D.);; 2 Hunan Provincial Key Laboratory of Emergency Safety Technology and Equipment for Nuclear Facilities, University of South China, Hengyang 421001, China

**Keywords:** demolition robot, attachment changing, error compensation

## Abstract

Attachment changing in demolition robots has a high docking accuracy requirement, so it is hard for operators to control this process remotely through the perspective of a camera. To solve this problem, this study investigated positioning error and proposed a method of error compensation to achieve a highly precise attachment changing process. This study established a link parameter model for the demolition robot, measured the error in the attachment changing, introduced a reference coordinate system to solve the coordinate transformation from the dock spot of the robot’s quick-hitch equipment to the dock spot of the attachment, and realized error compensation. Through calculation and experimentation, it was shown that the error compensation method proposed in this study reduced the level of error in attachment changing from the centimeter to millimeter scale, thereby meeting the accuracy requirements for attachment changing. This method can be applied to the remote-controlled attachment changing process of demolition robots, which provides the basis for the subsequent automatic changing of attachments. This has the potential to be applied in nuclear facility decommissioning and dismantling, as well as other radioactive environments.

## 1. Introduction

With the growing number of aging nuclear facilities and uncontrollable nuclear incidents, the ability to efficiently and safely treat and dispose of radioactive materials has become an important prerequisite for nuclear facility emergencies and nuclear facility decommissioning missions. Handling heavy radioactive materials, such as fuel rods or contaminated waste, as well as test, inspection, maintenance, and repair operations have been performed with robots for many years. A demolition robot is a kind of remote-controlled machine that is specially designed for working in dangerous environments and is widely applied in fields like nuclear facility emergencies and nuclear facility decommissioning [1,2]. Demolition robots are multifunctional, and their attachments, such as a bucket, grapple, metal shear, or breaker, can be changed based on different working conditions. Compared with the common method of changing construction machinery attachments manually, quick-hitch attachment changing of the demolition robot can be controlled remotely by the operator, which is safer and more efficient [3,4,5]. The BROKK company [6] from Sweden is one of the world’s leading manufacturers and has developed more than 15 types of demolition robots.

**Figure 1:**
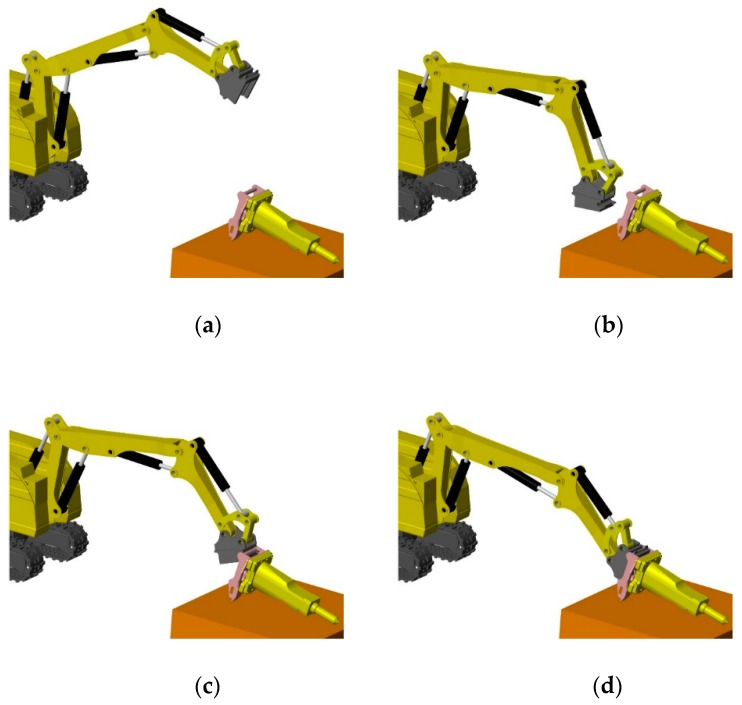
Attachment changing procedure: (**a**) initialization, (**b**) preparation, (**c**) range alignment, and (**d**) angle alignment.

Attachment changing of a demolition robot includes four procedures, as shown in Figure 1: First, initialization is carried out. In this stage, attachments begin to dock and the quick-hitch equipment is at the initial position and orientation. Second, preparation is done. At this stage, the quick-hitch equipment moves quickly to the preparation position. Third, range alignment occurs. The quick-hitch equipment moves to the dock spot and its coordinate origin overlaps with the attachment’s coordinate origin. Fourth, angle alignment is carried out. The quick-hitch equipment is rotated to make its coordinate frame overlap with the attachment’s coordinate frame. When this stage is finished, the hydraulic quick coupling (male) of the quick-hitch equipment connects with the hydraulic quick coupling (female) of the attachment. The hydraulic circuit is enabled and the quick-hitch equipment and the attachment lock up at the same time, thereby completing the attachment change. All movements of the robot are achieved through remote control of the operator. During the attachment changing procedure, the operator needs to observe the movement trajectory of the quick-hitch equipment from a close distance and multiple angles to meet the position accuracy requirements. When the working environment is hazardous [7,8], it is unsafe for the operator to enter the scene, and it is hard to complete remote attachment changing through cameras attached to the robot due to the narrow field of vision. If a visual system for attachment changing is provided, this problem can be solved and the working efficiency of the demolition robot can be improved.

A forward kinematics model needs to be established to develop the visual system of the demolition robot attachment changing. The coordinate transformation between the base frame and the end-effector frame is found using the Denavit–Hartenberg (D-H) model [9], which is common in motor-driven robotic applications. Because of the high machining accuracy and high precision encoder for position feedback, motor-driven robots have a high precision and a reliable stability level. The rotary encoder and potentiometer are used to measure the posture of the excavator robot end-effector, which can lead to automatic excavation [10,11,12,13,14]. However, the repeated positioning accuracy of hydraulic robots is worse than that of motor-driven robots due to the range of workspaces and mechanical structures. Positioning error can be compensated for by using the hand-eye calibration approach, which provides a robot calibration method when the base frame of a robot is difficult or even impossible to measure [15]. However, although the hand-eye calibration approach is suitable for the non-real-time calibration of small-size and high-precision industrial robots, the position error of the end-effector of the demolition robot is nonlinear and needs to be compensated for in real time. Attachment changing in the demolition robot requires positional preciseness, especially from the preparation stage to the range alignment stage. If the error in the position is above 5 mm, the task will not be completed. This study aimed to propose a method for real-time camera calibration that could compensate for the position error and achieve remote, long-distance control of attachment changing with the assistance of a 3D visualization model of the demolition robot. The main contributions of this paper are illustrated in the following points:

- The forward kinematics model of the BROKK 160 (BROKK MACHINES CO. LTD., Beijing, China) robot was established through measurement and calibration, and then error analysis of the motion trajectory of the robot’s end-effector was carried out. The results show that the large-sized robot driven by a hydraulic system could not achieve high-precision motion control through the conventional off-line calibration method.
- Based on this problem, a method of real-time error compensation for the attachment changing of large-size demolition robots was proposed. By introducing a reference coordinate system that was fixed near the dock spot of the robot quick-hitch equipment, this method was able to complete the coordinate transformation from the dock spot of the robot quick-hitch equipment to the dock spot of the attachment, thereby achieving the error compensation.
- Both indoor and outdoor experiments were carried out to verify this method. It was shown that the error compensation method proposed in this paper could reduce the level of error in the attachment changing process from the centimeter to the millimeter scale, thereby meeting the accuracy requirements.

## 2. Kinematics

**Figure 2:**
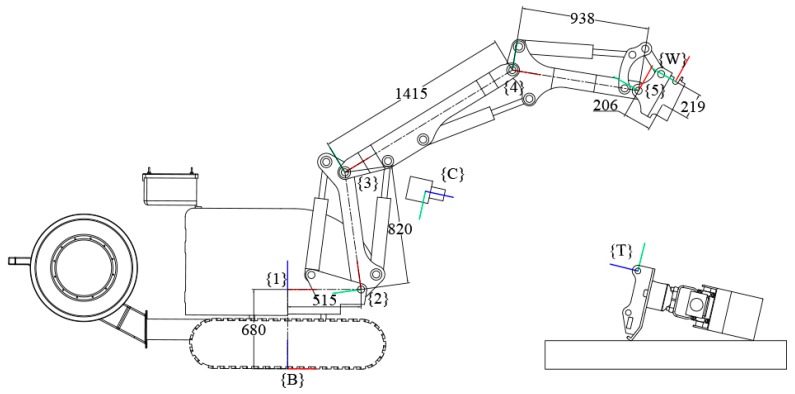
Coordinate frame of a BROKK 160 robot. {B} is the robot’s base coordinate frame, {W} is the quick-hitch dock spot coordinate frame, {C} is the camera coordinate frame, and {T} is the attachment dock spot coordinate frame (the red axis is the X-axis, the green axis is the Y-axis, and the blue axis is the Z-axis).

A demolition robot’s coordinate frame was established [16,17], as shown in Figure 2.

**Figure 3:**
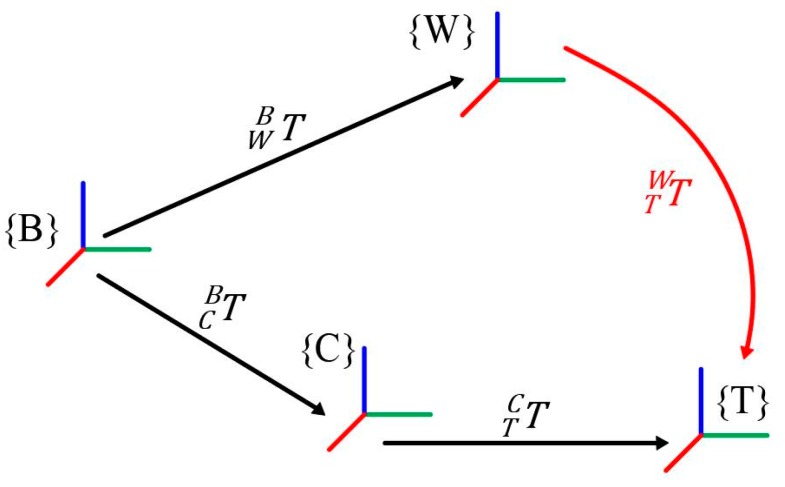
Coordinate frame transformation during the attachment changing process.

The key point of remote-control attachment changing lies in obtaining the attachment dock spot coordinate frame {T}, relative robot quick-hitch dock spot coordinate frame {W}, and homogeneous transformation matrix TTW, all while ensuring a small positioning error. TTW is expressed in Equation (1). The transformation of the coordinate frame during the attachment changing is shown in Figure 3.

(1) TTW=T−1WBTCBTTC

By measuring the BROKK 160 robot, then calibrating using the hand eye calibration method, the link parameters of the robot were obtained, as shown in Figure 2. Joints {1}, {2}, {3}, {4}, and {5} were revolute joints with rotation angles of *θ*_1_, *θ*_2_, *θ*_3_, *θ*_4_, and *θ*_5_, respectively. The lengths of the links were $l_{1}\;$= 680 mm, $l_{2}\;$= 515 mm, $l_{3}\;$= 820 mm, $l_{4}\;$= 1415 mm, $l_{5}\;$= 938 mm, $w_{x}\;$= 219 mm, and $w_{y}\;$= 206 mm.

Calculation of the homogeneous transformation matrix from the base coordinate frame {B} to the robot quick hitch equipment dock coordinate frame {W} is shown in Equation (2):

T1B=[cosθ1−sinθ100sinθ1cosθ100001l10001], T21=[cosθ2−sinθ20l200−10sinθ2cosθ2000001],

T32=[cosθ3−sinθ30l3sinθ3cosθ30000100001], T43=[cosθ4−sinθ40l4sinθ4cosθ40000100001],

T54=[cosθ5−sinθ50l5sinθ5cosθ50000100001], T5W=[100wx010−wy00100001].

TWB=T1BT21T32T43T54TW5

(2) $$=[\begin{array}{cccc} c_{2345}c_{1} & -s_{2345}c_{1} & s_{1} & \left(l_{2}+l_{3}c_{2}+l_{4}c_{23}+l_{5}c_{234}\right)c_{1}+w_{x}c_{2345}c_{1}+w_{y}s_{2345}c_{1}\\ c_{2345}s_{1} & -s_{2345}s_{1} & -c_{1} & (l_{2}+l_{3}c_{2}+l_{4}c_{23}+l_{5}c_{234})s_{1}+w_{x}c_{2345}s_{1}+w_{y}s_{2345}s_{1}\\ s_{2345} & c_{2345} & 0 & l_{1}+l_{3}s_{2}+l_{4}s_{23}+l_{5}s_{234}+w_{x}s_{2345}-w_{y}c_{2345}\\ 0 & 0 & 0 & 1 \end{array}],$$

where:

$$s_{1}=sin\theta_{1},\;c_{1}=cos\theta_{1},\;s_{23}=\sin\left(\theta_{2}+\theta_{3}\right),\;c_{2345}=\cos\left(\theta_{2}+\theta_{3}+\theta_{4}+\theta_{5}\right)\cdots$$

$$\begin{array}{c} l_{1}=680\;\mathrm{mm},\;l_{2}=515\;\mathrm{mm},\;l_{3}=820\;\mathrm{mm},\;l_{4}=1415\;\mathrm{mm},\\ l_{5}=938\;\mathrm{mm},\;w_{x}=219\;\mathrm{mm},\;w_{y}=206\;\mathrm{mm}. \end{array}$$

Through the above calculation, with the obtained values of *θ*_1_ to *θ*_5_, TWB was obtained. *θ*_1_ is the joint angle obtained by the chassis hydraulic motor, and the measurement of *θ*_1_ is complex. This study assumed that *θ*_1_ = 0. *θ*_2_ to *θ*_5_ are the values of joint angles created by the hydraulic cylinders. *θ*_2_ was measured directly using a digital inclination sensor. The length of the cylinder was measured using a linear displacement sensor, where the analog signal was converted to a digital signal using a 16-bit analog to digital converter, and the joint angles *θ*_3_ to *θ*_5_ were obtained via polynomial fitting of the length value of cylinder.

AprilTag 36h11 series tags [18,19] were stuck to the surface of the attachment. The positions and orientations of the tags were acquired using a BASLER avA1600-50gm camera (Basler Vision Technology Co., Ltd., Beijing, China), and then an attachment coordinate frame was obtained. Three AprilTag 36h11 tags were bundled and calibrated, and then the homogeneous transformation matrix TTC from camera {C} to the attachment dock spot coordinate frame {T} was obtained. The bundle calibration of the AprilTag 36h11 series tags, the recognition algorithm, and visualization were carried out using the apriltag_ros package [20,21].

## 3. Error Estimation

**Figure 4:**
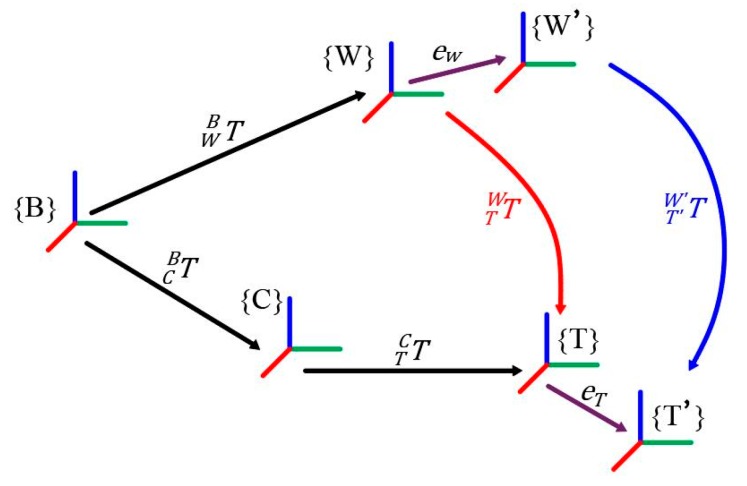
Errors during the attachment changing process.

Errors during the dock stage of BROKK 160 robot’s attachment changing process consisted of two parts, as shown in Figure 4: the error $e_{W}$ between the true value {W} of the BROKK quick-hitch equipment and the measured value {W′} and the error $e_{T}$ between the true value {T} of the attachment dock spot and the measured value {T′} according to the method shown in Section 2. The dock stage of the attachment changing requires the error of the distance between the true value TWB and the measured value TW′B′ to be less than 5 mm, and the relative error must be less than 0.1% (the maximum working distance of a BROKK 160 is 3.5 m).

$e_{W}$ is the error of TWB. The measurement of $e_{W}$ involved measuring the relative position and absolute angle using the laser range finder. The laser range finder was fixed on {W}, and then the joints (*θ*_1_ ≡ 0) of the robot were moved to let the laser project the mark point. The *θ*_2_ to *θ*_5_, distance, and inclination of the laser range finder were recorded, and then the previous steps were repeated to acquire more groups of data. The $e_{W}$ measurements are shown in Table 1.

**Figure 5:**
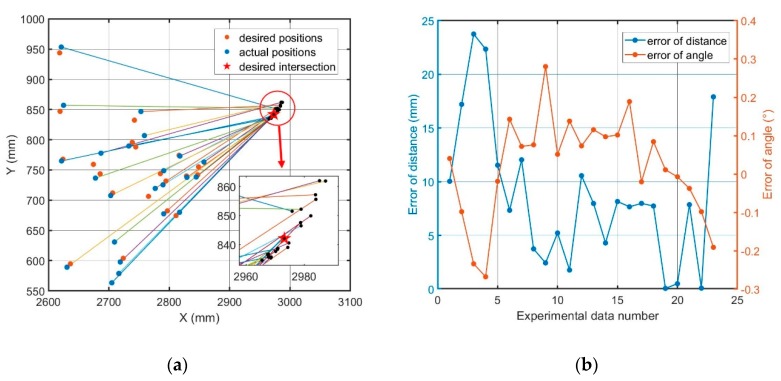
(**a**) Measured position error data of $e_{W}$. (**b**) Distance and angle error analysis of $e_{W}$.

There were 23 sets of measurement data in total. The joint angles in the data were used to calculate the position coordinates of the robot’s quick-hitch dock spot {W} using Equation (2), as shown by the blue dots in Figure 5a. At the same time, the rotation angle of {W} was determined. Because *θ*_1_ = 0, {W} only rotated around the Z-axis of the base coordinate frame {B}. This rotation angle is shown as R_Z_ in Figure 5a, where the lines represent the laser emitted by the laser range finder. The length of the line is equal to the value measured by the laser range finder, and the angle of the line is equal to R_Z_. The black dots in the partially enlarged figure are the positions of the calculated fixed mark points. As can be seen from Figure 5a, the positions of the marked points calculated through different sets of data did not overlap, indicating that an error $e_{W}$ existed between the measured value TW′B′ and the true value TWB.

The angle error of $e_{W}$ was obtained by comparing the calculated R_Z_ with the measured value from the laser range finder, as shown in Figure 5a by red and orange lines. The value angle RMS error was 0.13°, which is relatively small, and the influence of the angle error on attachment changing was found to be relatively small.

To calculate the position error of $e_{W}$, it was necessary to obtain the position of the marked point relative to the base coordinate {B}. Due to the large measurement error in the direct measurement, the least-squares method was adopted to calculate the desired intersection point of the laser. The sum of the distances between this intersection point and all marked points calculated through different sets of data was the minimum, as shown in Figure 5a. After the desired intersection point was obtained, the desired position of {W} was obtained using the measured distance and angle, as shown by the red dot in Figure 5a. The error of distance of {W} is shown by the blue line in Figure 5b. In the 23 sets of data, the RMS value of the distance error was 10.77 mm, the maximum error was 23.75 mm, and the minimum error was 0.07 mm. About 30% of the data in the seven sets met the requirement that the error should be less than 5 mm. The position error was caused by the measurement error of the sensor itself, the error and backlash of the robot connecting rod assembly, and the measurement error of the connecting rod size. For the BROKK 160 robot, it was difficult to keep the position error of $e_{W}$ below 5 mm.

## 4. Improvement

### 4.1. Reference Coordinates Frame

**Figure 6:**
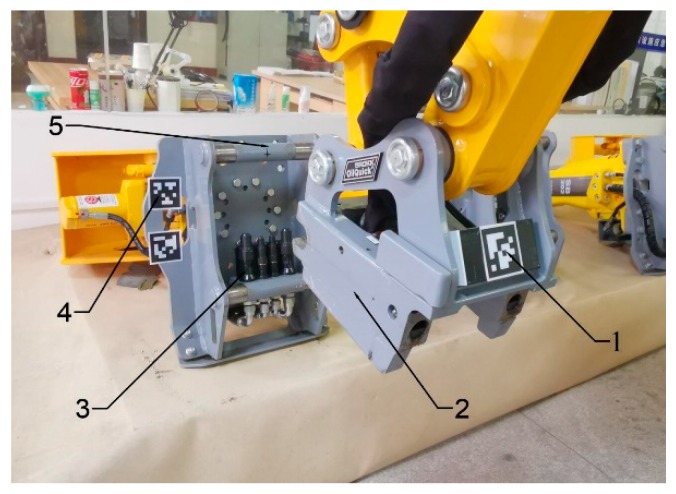
Introduction of the reference coordinate frame. (1) Tag of the reference coordinate frame {R}, (2) quick-hitch equipment, (3) hydraulic quick coupling (male), (4) tags of the attachment dock spot coordinate frame {T}, and (5) attachment dock spot.

Due to the existence of the ineliminable $e_{W}$, the TTW calculated using Equation (1) was too inaccurate to meet the requirements of attachment changing. In this section, by introducing the reference coordinate frame {R}, we describe our proposed method to solve TTW and compensate for the error $e_{W}$. The coordinate frame {R} was fixed on the quick hitch equipment, as shown in Figure 6.

The homogeneous matrix of {R} relative to {W} is TRW, and the homogeneous matrix of {R} relative to {C} is TRC. TTW can also be expressed using Equation (3):

(3) TTW=TRWT−1RCTTC.

**Figure 7:**
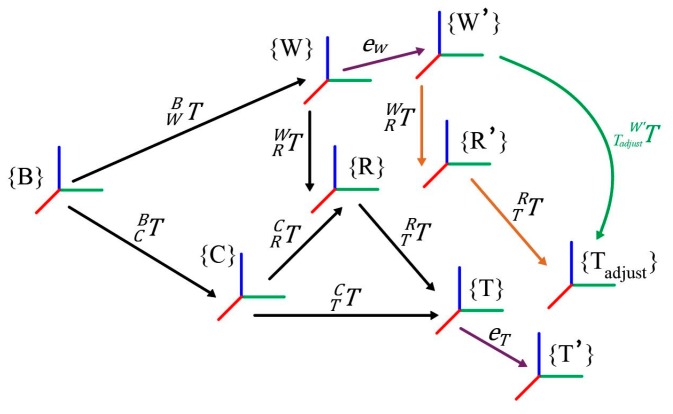
Error compensation in the process of attachment changing.

In the actual measurement process, the measured value {W′} of the dock spot of the robot quick-hitch equipment can only be obtained by collecting sensor data and introducing {*T_adjust_*}, as shown in Figure 7 and Equation (4):

TTadjustW′=TR′W′TTadjustR′

if: TR′W′=TRW, TTadjustR′=TTR

(4) TTadjustW′=TRWTTR=TRWT−1RCTTC=TTW

Equation (4) shows that when errors $e_{W}$ and $e_{T}$ cannot be eliminated, if the exact values of TRW and TTR can be obtained, the compensation coordinate frame {*T_adjust_*} of the attachment dock spot can be calculated. The equations TR′W′=TRW and TTadjustR′=TTR, which compensate for the camera coordinate frame {C_offset_}, were established.

Although {*T_adjust_*} did not overlap with the true value {T}, the error between TTadjustW′ and TTW was very small and met the docking accuracy requirements of the attachment changing of BROKK 160.

### 4.2. Offsetting the Camera Coordinate Frame {Coffset}

After the fourth stage of the attachment changing, namely angle alignment, {W} overlaps with the origin of {T}, the Y-axis of {W} overlaps with the Z-axis of {T}, and the X-axis of {W} overlaps with the Y-axis of {T}. Then:

(5) T4TW=Rotx(−90°)Rotz(−90°).

In Equation (5), the subscript 4 signifies the fourth stage, *Rot* means rotation, and the subscript *x* means rotation around the X-axis. The homogeneous transformation matrix of {C} to {R} was as follows:

T4RC=[1−0.00370.00450.01242−0.0054−0.88420.4671−0.02750.0022−0.4671−0.88421.480001].

The homogeneous transformation matrix of {C} to {T} was as follows:

T4TC=[10.0017−0.00400.003013−0.0006−0.8517−0.5240−0.2114−0.00430.5240−0.85171.7280001].

Equation (6) gives the constant value of TRW:

(6) TRW=T4TWTRT=Rotx(−90°)Rotz(−90°)T4−1TCT4RC

$$=\left[\begin{array}{cccc} 0.0075 & 0.5083 & -0.8611 & -0.2868\\ -0.0030 & 0.8612 & 0.5083 & 0.1152\\ 1.000 & -0.0052 & 0.0004 & 0.008229\\ 0 & 0 & 0 & 1 \end{array}\right].$$

The error compensation method in this paper can be understood as moving {R} and {T} to {R′} and {*T_adjust_*}. This is shown in Figure 7. Because the positions and orientations of {R} and {T} are obtained through camera coordinate frame {C}, {C} should be offset. The positions and orientations of {C} can be offset using Equation (7):

(7) TCoffsetB=TW′BTR′W′T−1R′Coffset=TW′BTRWT−1RC.

### 4.3. Error Compensation Algorithm

**Figure 8:**
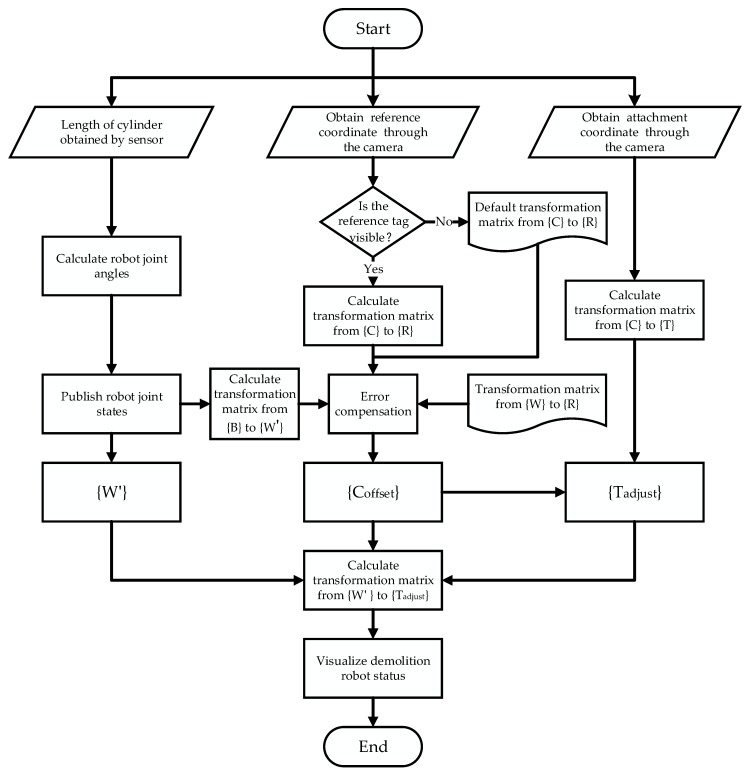
Block diagram of the error compensation algorithm.

As explained above, the error compensation algorithm goes through several steps. The block diagram of this algorithm is shown in Figure 8.

## 5. Experimental Research

### 5.1. Experimental Scene 1: Contrasting Experiment of Error Compensation

**Figure 9:**
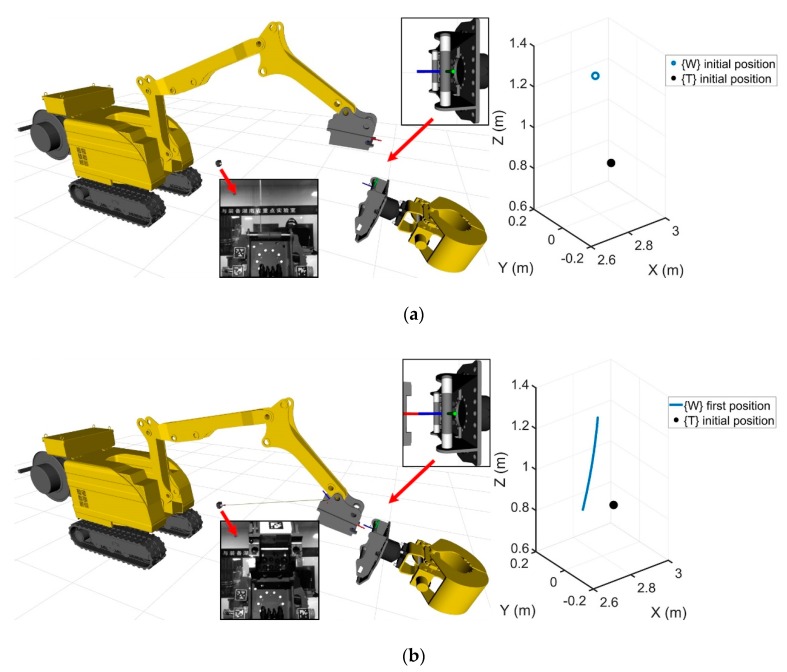

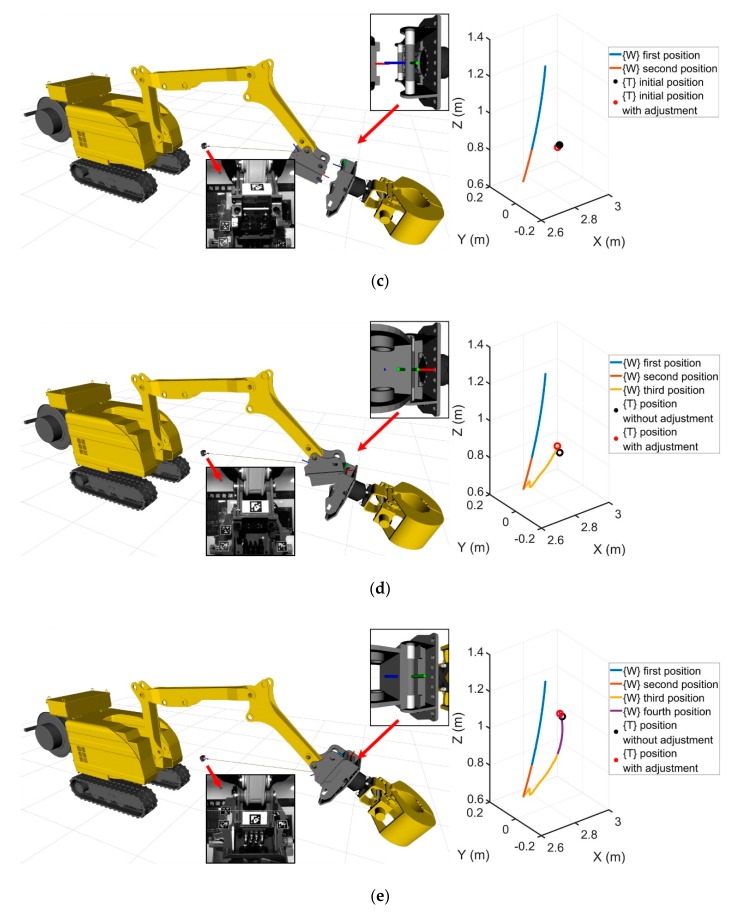

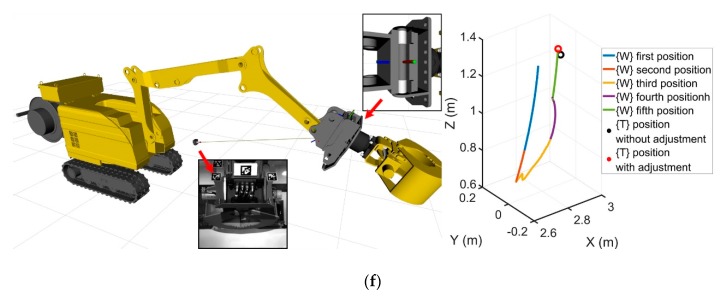
(**a**) Beginning of the experiment. (**b**) End of the first stage. (**c**) End of the second stage. (**d**) End of the third stage. (**e**) End of the fourth stage. (**f**) End of the experiment.

This study developed the brokk160 toolkit using the ros platform, which included a robot visualization program, a robot cylinder length data acquisition and joint angle conversion program, a Basler Pylon industrial camera driver, and the apriltag_ros program and real-time error compensation program described in Section 4. The experiment involved remotely controlling the BROKK 160 robot to complete the attachment changing process. The movement trajectories of {W} and {T} with and without the error compensation algorithm were recorded using rosbag. As shown in Figure 9, the experimental results were divided into five stages, lasting 52 s each, and 556 sets of motion track data were recorded.

#### 5.1.1. Initialization

In the experimental data, 0–9.6 s represents the first stage, which was the initial stage in the process of the tool head replacement. Figure 9a shows the experimental data at the beginning of the first stage. The left side of the figure shows the real-time 3D model of the robot (the oil cylinder model is omitted), the image obtained by the camera, and a magnified partial overlooking view at the attachment dock spot. On the right side of the figure, the initial 3D position coordinates of {W} (2.777, 0, 1.284) and {T} (2.863, 0.002, 0.826) are shown. At this stage, the reference coordinate frame {R} did not appear in the view of the camera {C}. The movement of {W} was controlled downward until the camera successfully captured the reference coordinate frame {R}, which was at a time of 9.6 s. The first stage ended at this point (and the second stage began at this point). Figure 9b shows the experimental data at the end of the first stage at a time of 9.6 s. The blue curve on the right is the movement track of {W} at this stage. {W} moved to (2.696, 0, 0.858) at 9.6 s.

#### 5.1.2. Preparation

In the experimental data, 9.6–12 s represents the second stage, which was the preparatory stage in the process of the robot tool head replacement. Figure 9b shows the experimental data when the starting point of the second stage is was taken as being 9.6 s. In the second stage, {W} continued downwards to find the appropriate docking path until 12 s. Figure 9c shows the experimental data at the end of the second stage at 12 s. It can be seen from the left side of the figure that the quick-change device of the robot was close to the tool head, and there was enough space to complete the docking between {W} and {T}. In Figure 9c, the orange curve on the right is the movement trajectory of {W} in the second stage.

#### 5.1.3. The Alignment Range

In the experimental data, 12–32 s represents the third stage, which was the docking stage in the process of attachment changing. Figure 9c shows the experimental data at the starting point of the third stage at 12 s. The third stage {W} moved to the upper-right side and entered the docking path. The distance between {W} and {T} became closer and closer until the docking was completed at 32 s. Figure 9d shows the experimental data at the end of the third stage (32 s). It can be seen from the real-time state of the 3D model of the robot and the magnified partial overlooking view that the position of {W} overlapped with that of {T}. After docking, the coordinates of the calibrated {T} position were (2.847, 0.002, 0.866), and the coordinates of the uncalibrated {T} position were (2.862, 0.004, 0.856). The yellow curve on the right of Figure 9d shows the movement trajectory of {W} in the third stage.

#### 5.1.4. The Angle Alignment and Operation Stages

In the experimental data, 32–40.2 s represents the fourth stage, which was the angle alignment stage in the robot attachment changing process, and 40.2–52 s represents the fifth stage, which was the operation stage after the completion of the attachment docking. Since the positions of {W} and {T} in the fourth stage and fifth stages always overlapped, they are discussed together. Figure 9d shows the experimental data at the starting point of the fourth stage at 32 s. In the fourth stage, the quick-hitch equipment rotated counter-clockwise around joint {5}, and the origins of {W} and {T} overlapped and rotated around the X-axis of {T} until 40.2 s, as shown in Figure 9e. The Y-axis of {W} overlapped with the Z-axis of {T}, and {W} was aligned with {T}. Finally, the process of attachment changing was completed. The calibrated position coordinates of {T} were (2.864, 0.002, 1.079) and the position coordinates of {T} without the error compensation were (2.878, 0.001, 1.058). As shown in Figure 9e, the purple curve on the right is the motion track of {W} in the fourth stage. 

At 40.2 s, the process of attachment changing was completed. The end device of the robot and the attachment conducted the hydraulic oil line and locked up through the hydraulic quick-hitch joint. At this time, the end device of the robot and the attachment were firmly connected, and {W} and {T} were always aligned. During the fifth stage, the end device and the attachment were controlled to rotate counter-clockwise around joint {3}. Figure 9f shows the experimental data in the fifth stage at 52 s, where the position coordinates of {W} were (2.894, 0, 1.336), the position coordinates with the error compensation of {T} were (2.895, 0, 1.338), and the position coordinates of {T} without the error compensation were (2.909, −0.001, 1.302). In Figure 9f, the green curve on the right is the motion track of {W} in the fifth stage.

#### 5.1.5. Discussion

**Figure 10:**
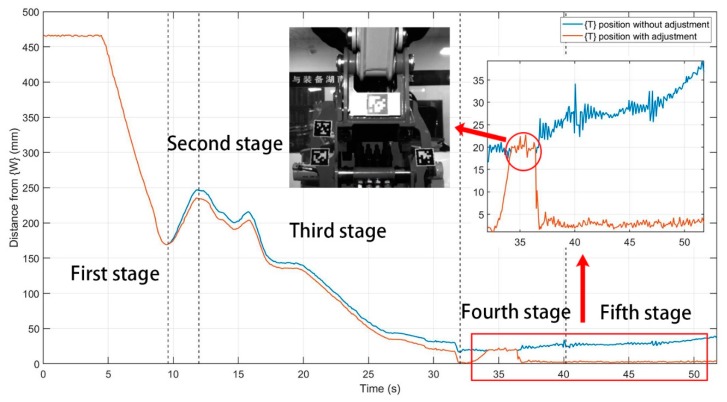
Error analysis of the experiment.

Since the reference coordinate system {R} did not appear in the view of the camera {C}, the position and orientation of {C} were default values that could be obtained by hand-eye calibration. Figure 10 shows the change in distance between {T} and {W} during the process of attachment changing. The blue line is the result obtained without the error compensation procedure, and the orange line is the result obtained with the error compensation procedure. From Figure 10, it can be seen that in the first stage, the two curves completely overlapped because there was no reference coordinate frame {R} for error compensation.

In the second half of the third stage, collisions and contacts occurred between the quick-hitch equipment of the robot and the attachment. {T} moved with the movement of {W}, and the two finally coordinated with each other and overlapped. As can be seen from Figure 10 of the third stage, the blue curve deviated from the compensated orange curve, which was moving closer to {W}. When the docking was completed, the distance between {T} and {W} decreased rapidly, and the attachment slid into the dock spot of the robot quick-hitch equipment. After docking, the distance between the compensated {T} and {W} was 1.68 mm, while the distance between {T} and {W} without the error compensation was 16.8 mm.

{W} and {T} were aligned in the fourth and fifth stages, and the relative distance between {W} and {T} was 0. Therefore, the effectiveness of the real-time error compensation described in Section 4 could be quantitatively evaluated in these two stages. Figure 10 is a partial enlarged view of the distance between {T} and {W} in the fourth and fifth stages with and without the error compensation. The data circled in red are the abnormal data of the orange curve from around 34–36 s, and the value was equal to the value of the blue curve. The reason for the abnormal data was that during the period of 34–36 s, the reference coordinate frame {R} was perpendicular to the light source, which generated a strong reflection, resulting in the camera misrecognizing the attitude of {R}. The real-time error compensation program in the BROKK 160 robot toolkit screened the attitude of {R}. Compared with the attitude in the previous frame, if the current attitude produced a large displacement and rotation, it was recognized as error data. The program output the default value of the camera {C} such that the orange curve overlapped with the blue curve in the period of 34–36 s. Apart from this interval, the distance between the adjusted {T} and {W} distances had nothing to do with the motion of {W}. 

The results of the contrast experiment of error compensation are shown in Table 2. The results analysis is listed in the following:

- In a safe scenario, the operator can observe the movement trajectory of the quick-hitch equipment from a close distance and multiple angles during the whole procedure until the task of demolition robot attachment changing can be finished.
- In a hazardous scenario, the operator remote attachment changing needs to be done through cameras, the third stage cannot be completed.
- In a hazardous scenario, the operator remote attachment changing is done through a visualization system. During the fourth and fifth stages, {W} and {T} are aligned and the relative distance between {W} and {T} should be 0. In this experiment, the distance without the error compensation from {W} to {T} increased along with the upward movement of {W}, with an average value of 26.68 mm, a minimum value of 16.65 mm, and a maximum value of 39.2 mm. The third stage could not be completed.
- The mean value of the distance with error compensation from {W} to {T} was 2.89 mm, the minimum value was 1.57 mm, and the maximum value was 4.53 mm. The task of the demolition robot attachment changing could be finished.

### 5.2. Experiment Scene 2: Attachment Remote Changing Indoors

This experiment was carried out in our laboratory under relatively ideal lighting conditions and on flat ground. The demolition robot was operated remotely and the crushing hammer disassembly and grab reloading was completed through a 3D visualization and error compensation system. A Video S1 of the whole experiment is available online at https://youtu.be/HnHOf0xLWXo.

### 5.3. Experiment Scene 3: Attachment Remote Changing Outdoors

This experiment was conducted outdoors under complex lighting conditions and on uneven ground. The demolition robot was operated remotely and the crushing hammer disassembly and grab reloading was completed through a 3D visualization and error compensation system. A Video S2 of the whole experiment is available online at https://youtu.be/ZM4Fkztawb4.

## 6. Conclusions

In this study, a method of real-time error compensation for the attachment changing of large-size demolition robots was proposed. This method converts the absolute error that is difficult to eliminate in the attachment changing process into a relative error, which is easy to control through real-time compensation of the camera coordinate frame. The experiments were carried out to verify that this method could reduce the level of error in the attachment changing process, and the maximum value of the position error was 4.53 mm, thereby meeting the accuracy requirements.

In this study, a visualization and error compensation system for remote demolition robot attachment changing was developed. The operator could achieve the attachment remote changing through this system, but the efficiency of this work still depends on the technical level and proficiency of the operator. In the next step in the dynamic robot model research, kinematic trajectory planning and hydraulic servo control research will be carried out to achieve automatic changing of the demolition robot attachment.

## Figures and Tables

**Table 1:** The $e_{W}$ measurement data.

*θ*_1_	*θ*_2_	*θ*_3_	*θ*_4_	*θ*_5_	Angle	Distance	Angle Error	Distance Error
0°	102.94°	−92.04°	−46.13°	19.18°	−16.0°	369 mm	0.04°	10.0 mm
0°	102.94°	−108.86°	−30.09°	69.31°	33.2°	248 mm	−0.10°	17.2 mm
0°	107.47°	−110.56°	−23.92°	40.54°	13.3°	235 mm	−0.23°	23.8 mm
0°	111.16°	−112.94°	−28.94°	46.49°	15.5°	310 mm	−0.27°	22.3 mm
0°	105.87°	−98.57°	−45.74°	37.67°	−0.8°	354 mm	−0.02°	11.5 mm
0°	94.88°	−94.49°	−45.74°	72.82°	27.6°	212 mm	0.14°	7.3 mm
0°	94.85°	−97.91°	−36.99°	74.88°	34.9°	152 mm	0.07°	12.0 mm
0°	91.12°	−81.95°	−67.79°	70.54°	12.0°	357 mm	0.08°	3.7 mm
0°	84.47°	−81.95°	−57.33°	89.73°	35.2°	177 mm	0.28°	2.4 mm
0°	84.36°	−78.59°	−68.45°	88.62°	26.0°	297 mm	0.05°	5.2 mm
0°	82.44°	−75.65°	−62.72°	79.20°	23.4°	171 mm	0.14°	1.8 mm
0°	78.20°	−67.83°	−78.58°	87.03°	18.9°	304 mm	0.07°	10.6 mm
0°	75.12°	−67.83°	−73.19°	97.49°	31.7°	210 mm	0.12°	8.0 mm
0°	73.92°	−67.83°	−70.07°	102.37°	38.5°	161 mm	0.10°	4.3 mm
0°	74.92°	−61.35°	−78.71°	76.24°	11.2°	239 mm	0.10°	8.1 mm
0°	74.86°	−71.24°	−70.81°	109.19°	42.2°	238 mm	0.19°	7.7 mm
0°	86.10°	−85.96°	−67.41°	103.59°	36.3°	418 mm	−0.02°	8.0 mm
0°	84.92°	−87.15°	−60.40°	106.25°	43.7°	345 mm	0.08°	7.7 mm
0°	93.32°	−96.09°	−51.75°	93.21°	38.7°	338 mm	0.01°	0.1 mm
0°	93.30°	−100.01°	−46.85°	99.36°	45.8°	368 mm	−0.01°	0.5 mm
0°	93.28°	−99.32°	−39.63°	91.71°	46.0°	233 mm	−0.04°	7.9 mm
0°	93.26°	−100.34°	−47.32°	100.60°	46.1°	387 mm	−0.10°	0.1 mm
0°	103.00°	−100.86°	−33.54°	33.99°	2.4°	231 mm	−0.19°	17.9 mm

**Table 2:** Results of the contrast experiment of error compensation.

Time	{W} Position	Error Compensation		Without Error Compensation	
		{T} Position	Distance from {W} to {T}	{T} Position	Distance from {W} to {T}
0 s	(2.777 m, 0 m, 1.284 m)	(2.863 m, 0.002 m, 0.826 m)	466.7 mm	(2.863 m, 0.002 m, 0.826 m)	466.7 mm
9.6 s	(2.696 m, 0 m, 0.858 m)	(2.863 m, 0.002 m, 0.826 m)	170.0 mm	(2.863 m, 0.002 m, 0.826 m)	170.0 mm
12.0 s	(2.646 m, 0 m, 0.705 m)	(2.853 m, 0.004 m, 0.816 m)	234.2 mm	(2.863 m, 0.002 m, 0.826 m)	245.8 mm
32.0 s	(2.849 m, 0 m, 0.865 m)	(2.849 m, 0.002 m, 0.866 m)	1.88 mm	(2.863 m, 0.002 m, 0.826 m)	16.93 mm
40.2 s	(2.863 m, 0 m, 1.077 m)	(2.864 m, 0.002 m, 1.079 m)	2.41 mm	(2.878 m, 0.001 m, 1.058 m)	24.93 mm
52 s	(2.894 m, 0 m, 1.336 m)	(2.896 m, 0.001 m, 1.338 m)	2.93 mm	(2.909 m, −0.001 m, 1.302 m)	36.81 mm
Min Value from 40.2 to 52 s	**―**	**―**	1.57 mm	**―**	16.65 mm
Max Value from 40.2 to 52 s	**―**	**―**	4.53 mm	**―**	39.20 mm
Mean Value from 40.2 to 52 s	**―**	**―**	2.89 mm	**―**	26.68 mm

## Supplementary Materials

The following are available online at https://youtu.be/HnHOf0xLWXo, Video S1: Experiment scene 2: Attachment remote changing indoors; https://youtu.be/ZM4Fkztawb4, Video S2: Experiment scene 3: Attachment remote changing outdoors.

## References

[1] Bogue R. (2011). Robots in the nuclear industry: A review of technologies and applications. Ind. Robot Int. J..

[2] Delmerico J., Mintchev S., Giusti A., Gromov B., Melo K., Horvat T., Cadena C., Hutter M., Ijspeert A., Floreano D. (2019). The current state and future outlook of rescue robotics. J. Field Robot..

[3] Kawatsuma S., Mimura R., Asama H. (2017). Unitization for portability of emergency response surveillance robot system: Experiences and lessons learned from the deployment of the JAEA-3 emergency response robot at the Fukushima Daiichi Nuclear Power Plants. ROBOMECH J..

[4] Buckingham R. (2012). Nuclear snake-arm robots. Ind. Robot Int. J..

[5] Burrell T., Montazeri A., Monk S., Taylor C.J. (2016). Feedback Control—Based Inverse Kinematics Solvers for a Nuclear Decommissioning Robot. IFAC Pap..

[6] Brokk Global. https://www.brokk.com.

[7] Takeyasu M., Nakano M., Fujita H., Nakada A., Watanabe H., Sumiya S., Furuta S. (2012). Results of environmental radiation monitoring at the Nuclear Fuel Cycle Engineering Laboratories, JAEA, following the Fukushima Daiichi Nuclear Power Plant accident. J. Nucl. Sci. Technol..

[8] Yoshida T., Nagatani K., Tadokoro S., Nishimura T., Koyanagi E., Yoshida K., Tadokoro S. (2014). Improvements to the Rescue Robot Quince Toward Future Indoor Surveillance Missions in the Fukushima Daiichi Nuclear Power Plant. Field and Service Robotics: Results of the 8th International Conference.

[9] Denavit J., Hartenberg R.S. (1995). Notation for lower-pair mechanisms based on matrices. J. Appl. Mech..

[10] Assenov E., Bosilkov E., Dimitrov R., Damianov T. (2003). Kinematics and dynamics of working mechanism of hydraulic excavator. Annu. Univ. Min. Geol..

[11] Fujino K., Moteki M., Nishiyama A., Yuta S. Towards Autonomous Excavation by Hydraulic Excavator—Measurement and Consideration on Bucket Posture and Body Stress in Digging Works. Proceedings of the 2013 IEEE Workshop on Advanced Robotics and Its Social Impacts.

[12] Chang P.H., Lee S.-J. (2002). A straight-line motion tracking control of hydraulic excavator system. Mechatronics.

[13] Yamamoto H., Moteki M., Shao H., Ootuki K., Yanagisawa Y., Sakaida Y., Nozue A., Yamaguchi T., Yuta S. (2010). Development of the autonomous hydraulic excavator prototype using 3-D information for motion planning and control. Trans. Soc. Instrum. Control Eng..

[14] Rezazadeh Azar E., McCabe B. (2012). Part based model and spatial–temporal reasoning to recognize hydraulic excavators in construction images and videos. Autom. Constr..

[15] Wu L., Ren H. (2017). Finding the Kinematic Base Frame of a Robot by Hand-Eye Calibration Using 3D Position Data. IEEE Trans. Autom. Sci. Eng..

[16] John C. (2017). Introduction to Robotics: Mechanics and Control.

[17] Peter C. (2017). Robotics, Vision & Control.

[18] Olson E. AprilTag: A Robust and Flexible Visual Fiducial System. Proceedings of the 2011 IEEE International Conference on Robotics and Automation.

[19] Wang J., Olson E. AprilTag 2: Efficient and Robust Fiducial Detection. Proceedings of the 2016 IEEE/RSJ International Conference on Intelligent Robots and Systems.

[20] Maximilian K., Acshi H., Edwin O. Flexible Layouts for Fiducial Tags. Proceedings of the IEEE/RSJ International Conference on Intelligent Robots and Systems (IROS).

[21] Brommer C., Malyuta D., Hentzen D., Brockers R. Long-Duration Autonomy for Small Rotorcraft UAS Including Recharging. Proceedings of the 2018 IEEE/RSJ International Conference on Intelligent Robots and Systems.

